# Mechanism and DNA-based detection of field-evolved resistance to transgenic Bt corn in fall armyworm (*Spodoptera frugiperda*)

**DOI:** 10.1038/s41598-017-09866-y

**Published:** 2017-09-07

**Authors:** Rahul Banerjee, James Hasler, Robert Meagher, Rodney Nagoshi, Lucas Hietala, Fangneng Huang, Kenneth Narva, Juan Luis Jurat-Fuentes

**Affiliations:** 1 Genome Science and Technology Graduate Program, University of Tennessee, Knoxville, TN 37996 USA; 2 Dow AgroSciences, Indianapolis, IN 46268 USA; 3 Center for Medical, Agricultural and Veterinary Entomology, Insect Behavior and Biocontrol Research Unit, USDA-ARS, Gainesville, FL 32608 USA; 4 Department of Entomology and Plant Pathology, University of Tennessee, Knoxville, TN 37996 USA; 5 Department of Entomology, Louisiana State University Agricultural Center, Baton Rouge, LA 70803 USA

## Abstract

Evolution of resistance threatens sustainability of transgenic crops producing insecticidal proteins from the bacterium *Bacillus thuringiensis* (Bt). The fall armyworm (*Spodoptera frugiperda*) is a devastating pest of corn in the Western Hemisphere initially controlled by transgenic Bt corn producing the Cry1Fa insecticidal protein (event TC1507). However field-evolved resistance to TC1507 was observed in Puerto Rico in 2007 and has subsequently been reported in a number of locations in North and South America. Early studies on Puerto Rico fall armyworm populations found that the resistance phenotype was associated with reduced expression of alkaline phosphatase. However, in this work we show that field-evolved resistance to Cry1Fa Bt corn in Puerto Rico is closely linked to a mutation in an ATP Binding Cassette subfamily C2 (ABCC2) gene that functions as a Cry1Fa receptor in susceptible insects. Furthermore, we report a DNA-based genotyping test used to demonstrate the presence of the resistant (*SfABCC2mut*) allele in Puerto Rico populations in 2007, coincident with the first reports of damage to TC1507 corn. These DNA-based field screening data provide strong evidence that resistance to TC1507 in fall armyworm maps to the *SfABCC2* gene and provides a useful molecular marker for detecting the *SfABCC2mut* allele in resistant fall armyworm.

## Introduction

Pest control efficacy and the potential for higher net returns have driven increasing adoption of transgenic crops producing insecticidal proteins from the bacterium *Bacillus thuringiensis* (Bt crops) during the past 20 years^1^. In the US, Bt corn and cotton adoption represent about 80% of the national acreage devoted to the two commodities^2^. This high level of adoption increases selection pressure for the evolution of resistance to Bt toxins in targeted pests. Currently, field evolved resistance resulting in control failures has been documented for three lepidopteran^3–7^ and one coleopteran^8^ species. Despite the importance of field resistance to the sustainability of Bt crops, little is known about the mechanisms involved, which in turn hinders the development of DNA-based technologies to monitor for resistance.

Alterations in any of the steps in the mode of action of the Bt insecticidal proteins can lead to resistance. Intoxication with Cry proteins from Bt includes processing to an activated toxin core by midgut proteases, binding to receptors on the brush border membrane of midgut cells, and formation of toxin pores that result in cell death by osmotic shock and collapse of the epithelial integrity, favoring the onset of septicemia and ultimately insect death (reviewed in ref. 9). The most commonly described resistance mechanism to Cry proteins from laboratory selection experiments is the alteration of toxin binding to midgut receptors^10^. While several proteins and glycolipids have been described as Cry toxin receptors in insects, resistance has been associated with mutations in cadherin and ATP binding cassette (ABC) genes, as well as to reduced expression of aminopeptidase (APN) or alkaline phosphatase (ALP) genes (reviewed in ref. 9). In cases of field-evolved resistance to Bt crops, differential splicing of cadherin transcripts was reported to associate with *Pectinophora gossypiella* (Saunders) resistance to Cry1Ac cotton in India^11^. Reduced expression of an alkaline phosphatase gene (*SfmALP2*) was associated with resistance to Cry1Fa corn in *Spodoptera frugiperda* (J. E. Smith) from Puerto Rico^12^. In this work, we report a mutation in an ABCC2 gene as linked to field resistance to Bt corn producing the Cry1Fa protein in *S. frugiperda* from Puerto Rico and describe a DNA-based method to monitor for resistance in field samples. Moreover, we demonstrate the feasibility of using this strategy on archived samples to provide a historical reconstruction of resistance evolution and dispersal.

## Results

### Resistance to Cry1Fa corn in *S. frugiperda* from Puerto Rico is linked to a mutation in the *SfABCC2* gene and not to reduced *SfmALP2* transcript levels

**Figure 1:**
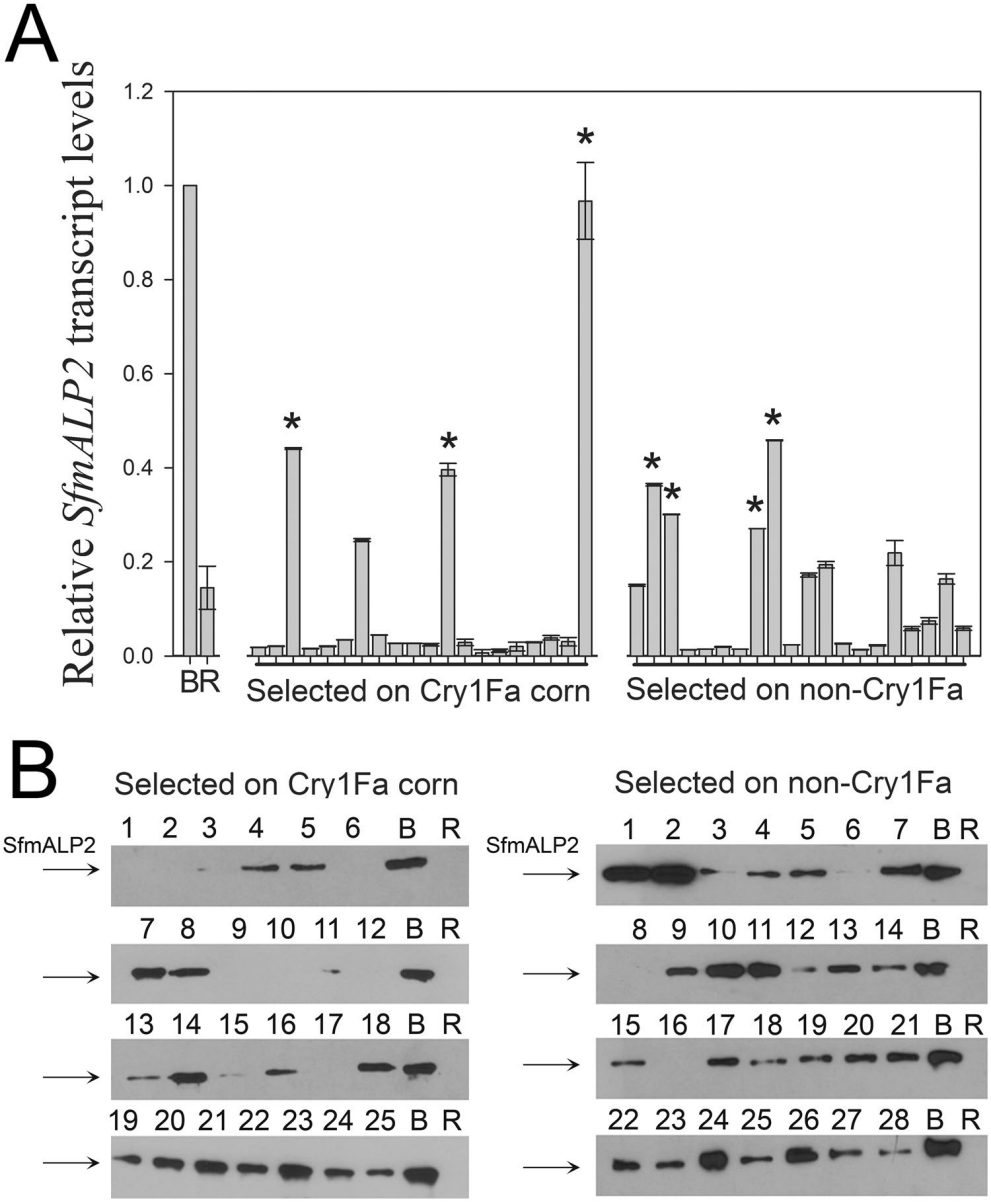
Lack of genetic linkage between reduced *SfmALP2* transcript (**A**) or protein (**B**) levels and resistance to corn event TC1507. A) Relative *SfmALP2* transcript levels in individual larvae from backcross families selected on Cry1Fa (TC1507) or non-transgenic isoline (non-Cry1Fa) corn, as indicated. The relative transcript levels were measured by quantitative real time PCR using the 2^(−∆∆Ct)^ method. Each bar represents the mean transcript levels and corresponding SE calculated from three technical replicates of individual insects relative to levels in the Benzon (susceptible, **B**) strain samples, which were considered as 1.0 for calculations. Asterisks denote significantly higher *SfmALP2* transcript levels when compared to 456LS3 (One Way ANOVA and post-hoc Holm-Sidak multiple comparison *P* < 0.01). (**B**) Western blots with BBMV proteins isolated from individual midguts of the backcross progenies selected on TC1507 or the non-transgenic isoline, as indicated, and probed with antisera detecting SfmALP2. Levels of detection for Benzon (B) and 456LS3 (R) are shown for relative comparison. Regions containing the cross-reactive SfmALP2 bands were cropped from full-length Western blots that are included as Supplementary Information (Fig. S4). Gel images were not enhanced or modified for contrast.

Previously we observed an association between reduced expression of a membrane-bound alkaline phosphatase gene (*SfmALP2*) and resistance to corn event TC1507 producing the Cry1Fa toxin in the 456LS3 strain of *S. frugiperda* from Puerto Rico^12^. However, genetic linkage analyses (Fig. S1) did not support co-segregation of reduced SfmALP2 levels and resistance to corn event TC1507 in the 456LS3 strain (Fig. 1). Quantification analyses found nearly equal proportions of individuals with *SfmALP2* transcript levels similar to or lower than that of 456LS3 larvae in survivors from TC1507 (17/20) and non-Bt (16/20) treatments (Fig. 1A). Similarly, survivors of both treatments showed a wide range and substantial overlap in SfmALP2 protein levels (Fig. 1B). These results contrast with the expectation of unambiguous differences between the two treatments in the distribution of the genetic trait associated with the resistance phenotype (Fig. S1), a strong indication that ALP is not the primary determinant for survival on TC1507. However, there were still indications of a correlation between ALP levels and Cry1Fa resistance. The survivors of the TC1507 treatment were less likely than their non-Cry1Fa treatment counterparts to express SfmALP2 transcript levels above baseline (>0.1) (Fig. 1A) or show SfmALP2 protein levels greater than the Benzon strain (susceptible parents) control (Fig. 1B).

**Figure 2:**
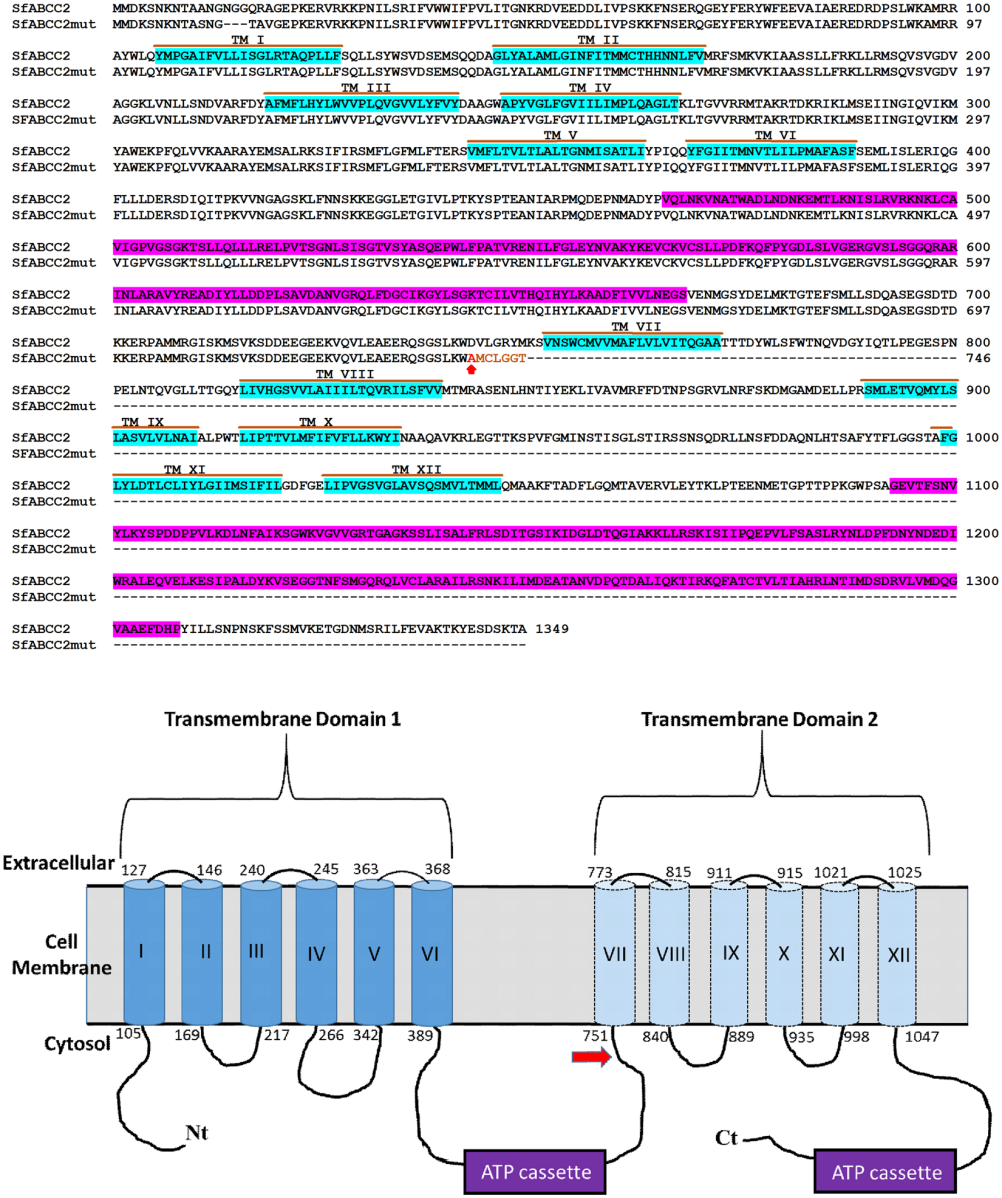
Alignment of the translated SfABCC2 and SfABCC2mut protein sequences (upper panel) and representative diagram of the predicted SfABCC2 protein structure and location of the mutation in *SfABCC2mut* (lower panel). In the sequence alignment the transmembrane regions are highlighted in blue, and the predicted ATP binding cassettes are highlighted in purple. The lower panel diagram is not drawn to scale and includes the localization of the ATP binding cassettes (purple “ATP cassette” boxes) and the approximate location of the mutation in *SfABCC2mut* (red arrow). Numbers indicate the amino acid residue at that location. The dotted lines and lighter blue color for the Transmembrane Domain 2 represent the predicted region missing in the truncated SfABCC2mut protein.

Transcriptomic profiling of putative Cry1Fa receptors in susceptible and a TC1507-resistant strain of *S. frugiperda* originated by Dow AgroSciences from Puerto Rico collections, detected differences for the *SfABCC2* gene between the strains. A number of conservative single nucleotide polymorphisms, a 9 b deletion of bases 39–47, and a 2 bp (GC) insertion at position 2,218 were detected in the cDNA from the resistant compared to the susceptible strain (Fig. S2). The GC insertion was confirmed by sequencing both cDNA and genomic DNA templates to be present in the 456LS3 but not in the Benzon strain, and thus we named the allele containing the insertion as *SfABCC2mut*. This insertion induces a frameshift and a premature stop codon at amino acid 746 (Fig. 2, upper panel), resulting in production of a truncated protein predicted to miss the whole second transmembrane domain in the expected protein structure, including one of the ATP binding cassettes (Fig. 2, lower panel).

A custom Taqman SNP genotyping assay was developed to detect the *SfABCC2mut* allele and used to test for its genetic linkage with resistance to TC1507 corn in the 456LS3 strain. As expected from Mendelian transmission of a single recessive resistance allele (Fig. S1), all the genotyped 456LS3 parents were homozygous for the *SfABCC2mut* allele, while the allele was not detected among parents from the Benzon (susceptible) strain. All genotyped F1 progeny from Benzon × 456LS3 crosses were identified to be heterozygous for the *SfABCC2mut* allele (Table 1). In the backcross subfamily selected with the non-transgenic corn isoline, there was no significant difference (two-tailed *P*-value = 2.469; χ^2^ = 0.1161) between the proportion of expected (1:1) and observed (30:19) heterozygous and homozygous individuals, respectively, for the *SfABCC2mut* allele (Table 1). In contrast, the proportion of observed heterozygous to homozygous individuals (8:42) among the survivors of the backcross subfamily exposed to corn event TC1507 highly significantly diverged (two-tailed *P*-value < 0.0001; χ^2^: 23.120) from the expected 1:1 ratio, supporting co-segregation.

**Table 1:** Detection of *SfABCC2mut* allele in strains and crosses testing linkage with resistance to corn event TC1507 in the 456LS3 strain of *S. frugiperda*.

Strain/Cross	SS	Sr	rr	Total
Benzon	29	—	—	29
456LS3	—	—	31	31
F1 – Ben × 456LS3	—	25	—	25
Backcross on TC1507	—	8	42	50
Backcross on non-Bt	—	30	19	49

### SfABCC2, but not SfABCC2mut, serves as a functional shared Cry1A/Cry1Fa receptor in cultured insect cells

The full-length cDNAs encoding SfABCC2 (accession number KY489760) and the truncated SfABCC2mut (accession number KY489761) proteins were cloned and expressed in cultured Sf9 cells, as confirmed by Western blotting (Fig. S3A). In replicated experiments, the detected recombinant protein bands appeared consistent with the predicted size for SfABCC2 (151.6 kDa) and SfABCC2mut (84.6 kDa) proteins and were detected in the membrane fraction. Relatively lower levels of recombinant protein were detected in cells producing SfABCC2mut, but increasing levels of SfABCC2mut did not result in augmented Cry1Fa binding (Fig. S3A).

**Figure 3:**
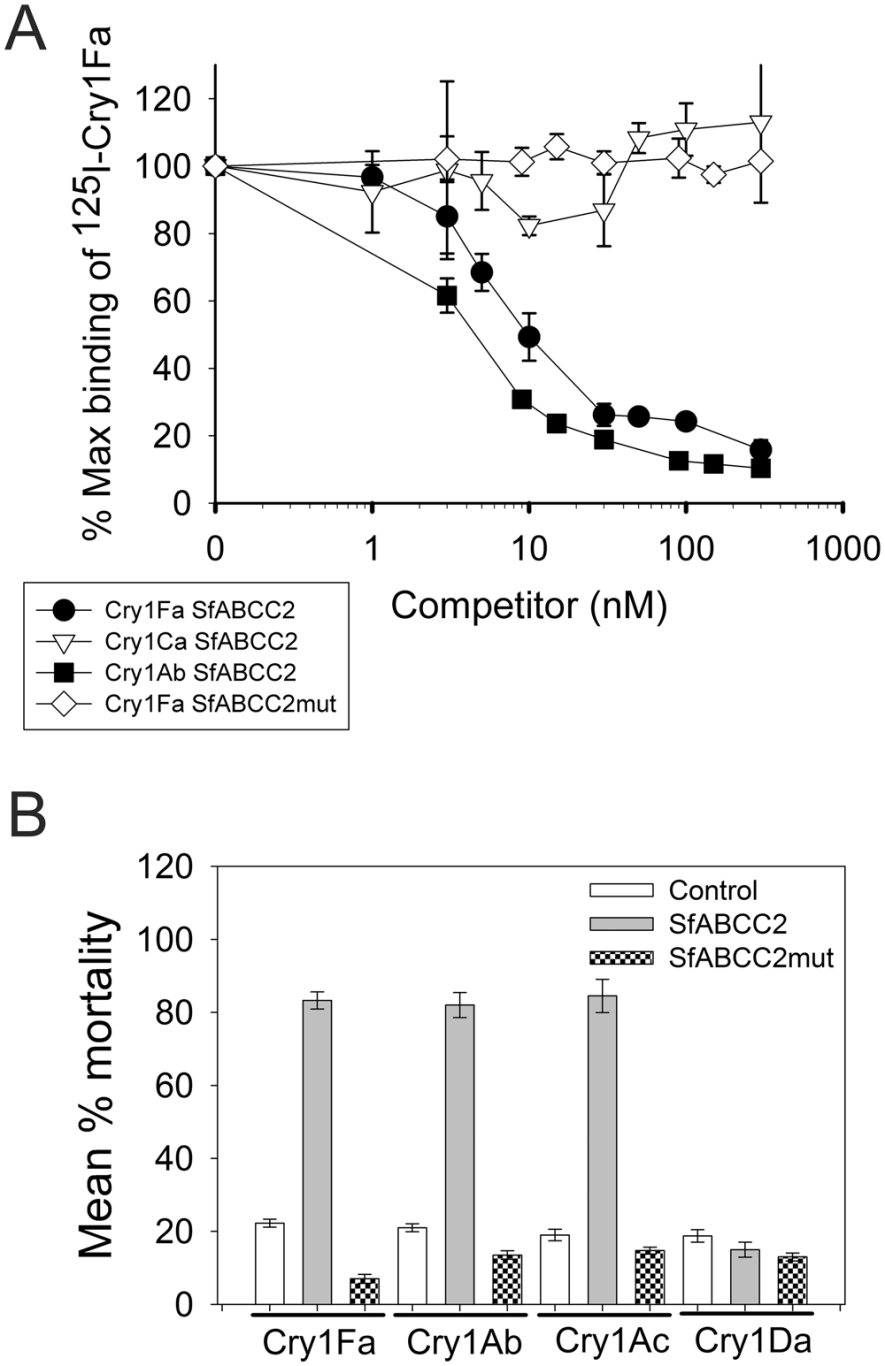
Competition of Cry1Fa binding (**A**) and cytotoxicity (**B**) in cells producing recombinant SfABCC2 or SfABCC2mut proteins. Curves in (**A**) display competition of ^125^I-Cry1Fa binding to Sf9 cells producing the SfABCC2 or SfABCC2mut proteins, as indicated in the figure legend, by Cry1Fa (black circles and open diamonds), Cry1Ab (black squares) or Cry1Ca (open inverted triangles) as unlabeled competitors. Data shown are the means and corresponding standard errors from two independent experiments performed in duplicate (N = 2). (**B**) Cell mortality in cultures exposed to 0.1 µg/ml of Cry1Fa, Cry1Ab, Cry1Ac, or Cry1Da activated toxins. Mortality in cells infected with empty bacmid (control), cells producing SfABCC2, and cells producing SfABCC2mut was determined after incubation for 24 hours using trypan blue exclusion. Data shown are the means and corresponding standard errors from two independent experiments (N = 2).

Results from qualitative binding assays (Fig. S3B) confirmed specific binding of radiolabeled Cry1Fa to Sf9 cells producing SfABCC2, but not to the control cells transfected with empty vector or cells producing SfABCC2mut. In competition binding assays, radiolabeled Cry1Fa bound with high affinity (*K*_*d*_ = 1.26 ± 0.31 nM) to Sf9 cells producing SfABCC2, while no specific Cry1Fa toxin binding was detected to cells producing SfABCC2mut (Fig. 3A). Moreover, Cry1Ab bound to cells producing SfABCC2 with significantly higher affinity (*K*_*d*_ = 0.20 ± 0.03 nM) but similar binding site concentration (1.06 ± 0.09 nmol/mg) than Cry1Fa (1.30 ± 0.13 nmol/mg). In contrast, Cry1Ca did not displace Cry1Fa binding (Fig. 3A), supporting that the former protein does not bind to the shared Cry1A-Cry1Fa sites on SfABCC2.

Binding of Cry1Fa to Sf9 cells producing SfABCC2 was conducive to toxicity, as 80% mortality was observed after treatment with 0.1 µg/ml (1.67 nM) of Cry1Fa (Fig. 3B). In contrast, mortality was significantly lower (One Way ANOVA and post-hoc Holm-Sidak pairwise comparison, *P* < 0.01) in Sf9 cells producing SfABCC2mut (17%) or in cells transformed with the empty vector (24%). Similar results were obtained when exposing Sf9 cells producing SfABCC2 to Cry1Ab or Cry1Ac (Fig. 3B). Since Sf9 cells are highly sensitive to Cry1Ca^13^, we tested susceptibility to Cry1Da as a negative control. This protein is active against *S. frugiperda* ^14^, but it was inactive against any of the transformed or control Sf9 cells tested (Fig. 3B).

### Historic-geographic detection of the *SfABCC2mut* allele in field populations of *S. frugiperda*

**Figure 4:**
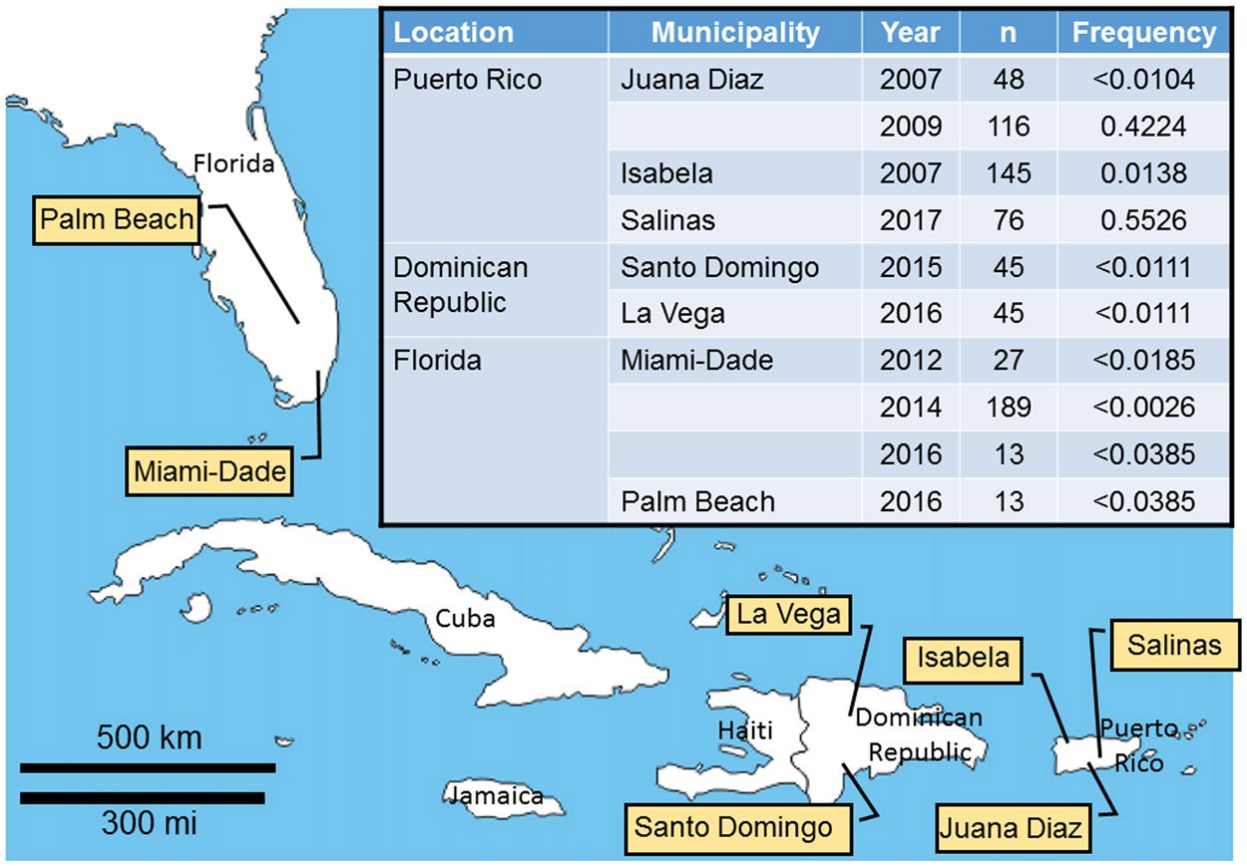
Frequency of the *SfABCC2mut* allele in archived and recent *S. frugiperda* field collections from Puerto Rico, Florida and Dominican Republic. Shown in the map are the collection locations. The table inset presents the frequency of the *SfABCC2mut* allele depending on location and year of collection, as well as the number of individuals genotyped (n). Frequency is calculated based on data in Table S1 using the Hardy-Weinberg equilibrium model as described in the Materials and Methods section. The map shown was modified from http://www.d-maps.com/carte.php?num_car=1384&lang=en.

Migratory movement of *S. frugiperda* was suggested to explain cases of *S. frugiperda* resistance to TC1507 in Florida, Georgia and North Carolina^7^. To test this hypothesis, we genotyped archived *S. frugiperda* genetic materials from collections in Puerto Rico (2007, 2009, and 2017), Florida (2012, 2014 and 2016) and Dominican Republic (2015 and 2016) using the custom Taqman assay (Fig. S5) and confirming selected samples by DNA sequencing (data not shown). Genotyping of samples from Puerto Rico (Fig. 4) confirmed that the *SfABCC2mut* allele was present in low frequency in 2007 in the Isabela region (0.0138, n = 169), when field control failures were starting to be commonly reported^5^. Resistance spread to Juana Diaz where the allele was found at a relatively high frequency (0.4224, n = 116) in 2009. In the most recent collection of *S. frugiperda* larvae in Puerto Rico (Salinas municipality, close to Juana Diaz), the frequency of the resistance allele was the highest detected (0.5526, n = 76). In contrast, we did not detect the *SfABCC2mut* allele in any of the 246 moths genotyped from different counties in Florida (collected in 2012, 2014 or 2016), or the 90 from two municipalities in Dominican Republic (2015 and 2016).

## Discussion

Extensive use of transgenic Bt crops for more than two decades has led to evolution of field-evolved resistance in selected lepidopteran pests, threatening sustainability of the technology and resulting in pest control failures at diverse geographies^3–6^. The need to hinder evolution of resistance to Bt crops makes it imperative to understand the molecular mechanisms involved. In this study we report SfABCC2 as a functional Cry1Fa/Cry1A receptor in *S. frugiperda* and identify a mutation tightly linked to field-evolved resistance to transgenic corn event TC1507 producing the Cry1Fa toxin (Herculex I®) in *S. frugiperda* populations from Puerto Rico. We also demonstrate the detection of this mutant allele (*SfABCC2mut*) in current and archived field-collected *S. frugiperda* genetic samples. Genotyping data support that he *SfABCC2mut* allele emerged and exists at high frequency in Puerto Rico but it is not detected in migratory *S. frugiperda* destinations in the Caribbean and Florida.

Data from linkage tests in this study did not support a previous hypothesis that reduced *SfmALP2* transcript and protein levels were linked with resistance to TC1507 corn in 456LS3 larvae^12^. This discrepancy could be explained by the *SfABCC2mut* allele influencing expression of *SfmALP2*. While further work would be needed to test this hypothesis, silencing of the *PxABCC2* gene in *Plutella xylostella* (L.) larvae did not affect expression of the *PxmALP* gene orthologous to *SfmALP2* ^15^. Alternatively, it is possible that reduced SfmALP2 levels may increase resistance levels in individuals that are already homozygous for the *SfABCC2mut* allele. In support of this hypothesis, we did not detect larvae with relatively high SfmALP2 levels among survivors of exposure to TC1507 corn. Consequently, larvae displaying lower levels of *SfmALP2* expression were in higher proportion among survivors of exposure to Cry1Fa corn compared to the treatment with non-transgenic isoline, which may help explain the reported association between lower ALP levels and resistance to Cry1Fa^12^.

Mutations in ATP binding cassette genes have been reported to be linked to resistance against Cry1^16–19^, Cry2^20^, and Cry3^21^ toxins. In the case of *Spodoptera* spp., resistance against Cry1Ca in a strain of *S. exigua* (Hübner) was linked to a mutation in an ABCC2 gene^22^. Importantly, silencing of expression of that ABCC2 gene in *S. exigua* larvae led to reduced susceptibility to Cry1Ca as well as Cry1Ac. This information is in discrepancy with our findings identifying SfABCC2 as a shared Cry1Fa-Cry1A receptor that is not recognized by Cry1Ca in *S. frugiperda*. Moreover, Cry1A and Cry1Ca toxins have been demonstrated not to share binding sites in *Spodoptera* spp.^12, 23^, and the 456LS3 strain is resistant to Cry1Fa, Cry1Ac and Cry1Ab but remains susceptible to Cry1Ca and displays unaltered Cry1Ca binding^12^. These discrepancies suggest that silencing of ABCC2 genes in *S. exigua* may have unintended effects on expression of non-target genes affecting Cry1Ca mode of action, or that Cry1Ca and Cry1A toxins do bind to the same ABCC2 proteins but recognize different binding epitopes on these proteins. In any case, we did not detect specific Cry1Fa toxin binding or toxicity against Sf9 cells producing SfABCC2mut. Considering currently proposed models^24^, it is plausible that lack of an available SfABCC2 in an open state prevents binding of Cry1Fa toxin oligomers and lateral movement into the membrane for pore formation.

While a small set of insects were tested, results from genotyping *S. frugiperda* genetic materials allow for estimations of allele frequencies in the collected insects, which should reflect population frequencies. Results from genotyping archived materials support that the *SfABCC2mut* allele was present in Puerto Rico as early as 2007. The time course of increased *SfABCC2mut* allele frequency from 2007 to 2009 corresponds with increased observations of TC1507 damage by *S. frugiperda* in Puerto Rico^5^. This increase after only two years (2007-2009) occurred even after corn event TC1507 was withdrawn from the local market^25^, suggesting that the presence of TC1507 was not necessary to maintain the allele in field populations. These observations and the consistently high levels of resistance detected in Puerto Rico 5 years after the initial resistance report^26^, are in agreement with the lack of significant fitness costs for the *SfABCC2mut* allele detected under laboratory conditions^27^.

Recent studies reported Cry1F resistance allele frequencies in Florida, Louisiana, and North Carolina at between 10–29%, suggesting widespread regional establishment of the resistance trait at a substantial frequency^7, 28^. The ability to detect the *SfABCC2mut* allele by PCR provides a new genetic marker for monitoring fall armyworm migratory movements, with the current data providing no evidence for substantial northward migrations from Puerto Rico into Florida. The *SfABCC2mut* allele frequency in Puerto Rico populations has not greatly vary since resistance was examined in 2009 and ranged from 42% in 2009 to 55% in 2017. We compared this to frequencies in the Dominican Republic, an island that lies less than 150 km from Puerto Rico and a likely intermediate stop for any fall armyworm migration from Puerto Rico to Florida. The *SfABCC2mut* allele was not detected in collections from 2015–2016 (n = 90) indicating an allele frequency of <0.011%. A similar result was obtained in our survey of 246 specimens at two major corn regions in southern Florida where the SfABCC2mut mutation was again not detected, suggesting a frequency <0.004%. These findings suggest that the SfABCC2mut mutation may not be the Cry1Fa resistance allele reported in 29% of fall armyworm tested in Florida {Huang, 2014 #17260}{Vélez, 2013 #16799}. Additional genotyping efforts will be necessary to assess the genetic relationship between the Bt-resistance phenotypes observed in the United States and Puerto Rico.

Considering that the 456LS3 strain was generated from individuals collected from the same southern part of Puerto Rico (Santa Isabel and Juana Diaz) where resistance to corn event TC1507 was originally detected^5, 29^ and our genotyping results, we hypothesize that *SfABCC2mut* was responsible for the original field resistance reports in Puerto Rico. Availability of sensitive DNA-based tests detecting this field-evolved resistance allele enables acquisition of data conducive to development of predictive models for dispersal of resistance to transgenic corn. Current work is focused on identifying the resistance allele in *S. frugiperda* populations from Florida and determining the frequency of resistance across diverse migratory locations to explain the apparent containment of the *SfABCC2mut* allele to Puerto Rico.

## Materials and Methods

### Insect strains

Eggs of a susceptible (Benzon) *S. frugiperda* strain were acquired from Benzon Research Inc. (Carlisle, PA). The Cry1Fa-resistant 456 strain^29^ cross-resistant to Cry1Ab and Cry1Ac^30^ was used as parental line to generate strain resistant strain 456LS3 by sequentially crossing^29^ to the Benzon strain and selecting the F2 with leaf material of corn event TC1507 for 5 days. This procedure was repeated four times to increase isogenicity of the strains. Larvae were reared in the laboratory with selection for 5 days after hatching with corn event TC1507 producing Cry1Fa toxin (strain 456LS3) or with the non-transgenic isoline 2T777 (Benzon), and then transferred onto artificial diet (BioServ) to complete larval development. Insect rearing was as described elsewhere^12^.

### Toxin purification and labeling

The Cry1Fa, Cry1Ca, and Cry1Da proteins were produced in recombinant *Pseudomonas fluorescens* strains and purified as described elsewhere^31^. The Cry1Ab protein was produced in a recombinant *Escherichia coli* strain and purified as described previously^12^. Purified Cry1Fa toxin (25 µg) was labeled with 0.5 mCi of iodine-125 (Perkin Elmer) as described elsewhere^32^. Purity of the labeled toxin was assessed by SDS-10%PAGE followed by autoradiography.

### Cloning and expression of SfABCC2 and SfABCC2mut

Total RNA was isolated from 200 mg of 1st-2nd instar *S. frugiperda* larvae using the RNAqueous midi kit (Thermofisher) following the manufacturer’s instructions. The Dynabeads mRNA isolation kit (Thermofisher) was used to purify mRNA from 75 μg of total RNA, and first strand cDNA was synthesized from mRNA using SuperScript III first strand cDNA synthesis kit (Invitrogen). Primers containing BamHI sites were designed to amplify the *SfABCC2* sequence identified as most homologous to other lepidopteran ABCC2 genes in a custom *S. frugiperda* transcriptome generated at Dow AgroSciences. Forward primer (5′ to 3′) was SfABCC2For2 5′ GGATCCACCATGATGGACAAATCTAATAAA 3′, and reverse primer SfABCC2Rev2 was 5′ GGATCCTTAGTGATGGTGGTGATGGTGAGCGGTTTTGGAATCAC 3′. Amplification was performed using Phusion Hot Start II Polymerase (New England Biolabs) with 30 cycles of denaturation at 98 °C for 10 sec, annealing at 50.8 °C for 30 sec, and extension at 72 °C for 2.5 minutes. The amplicon was purified and cloned into pBAcPAK9 using the engineered BamHI sites, and a correct clone was confirmed by restriction analysis and used to generate endotoxin-free transfection quality DNA. Purified cDNA from a Cry1Fa-resistant *S. frugiperda* strain collected by Dow AgroSciences in Puerto Rico (same origin as 456LS3) was used as template to clone *SfABCC2mut*, following the procedure outline for *SfABCC2*. A 6X-His tag was engineered into the cDNAs to add the tag to the C-terminus of both the wild type and the truncated SfABCC2mut proteins for detection. The full-length cDNA sequences have been deposited in GenBank with accession numbers KY489760 (*SfABCC2*) and KY489761 (*SfABCC2mut*).

For recombinant virus production, Sf9 cells (in Sf900III SFM) were seeded into a 12-well plate at 5 × 10^5^ cells/well and allowed to attach at 27 °C for 1 hour. The following mixes were made in polystyrene tubes; 41 μl sterile H_2_O, 2.5 μl pBacPAK9/SfABCC2 (or pBacPAK9/SfABCC2mut), 2.5 μl BestBac2.0 linearized parental DNA, 4 μl Cellfectin II. After incubation at room temperature for 20 minutes, each transfection mix was used to transfect Sf9 cells. After six days at 27 °C, the virus in the media were collected and clarified from cell debris by centrifugation. To amplify the virus, flasks of Sf9 cells seeded at 2 × 10^6^ cells/ml were transfected as above and virus collected after 48 hours by filtration of the media. For titer determination, a 0.5 ml sample of each virus was sent to Expression Systems Titer service (Davis, CA).

Recombinant virus was added at a multiplicity of infection (moi) of 5 to shake cultures of Sf9 cells seeded at 1 × 10^6^/ml, and cultures were incubated overnight at 27 °C and 135 rpm for 24 hours. After incubation, cells were collected by centrifugation and used for cytotoxicity assays or stored at −80 °C until used for Western blotting or binding assays.

### Genetic linkage

Materials from the same backcross experiment (Fig. S1) were used to test co-segregation of SfmALP2 down-regulation or *SfABCC2mut* with resistance to TC1507 corn. Adults from the 456LS3 and Benzon strains were crossed to generate a heterozygous F1 generation. Males of this F1 generation (n = 20) were backcrossed with 456LS3 females (n = 20) to generate an F2 generation, which was divided into two subfamilies as neonates. Subfamily A (n = 240) was exposed to Cry1Fa corn event TC1507, while subfamily B (n = 238) was reared on the non-transgenic corn isoline. After five days, survivors (n = 117 from subfamily A and n = 205 from subfamily B) were put on artificial diet (BioServ) to allow for completion of their developmental process and then guts dissected to purify BBMV, total RNA or genomic DNA, which were used for protein detection, quantitative PCR or Taqman genotyping (25–50 individuals per experiment from each subfamily). Statistical deviation of observed from expected proportions of heterozygote and homozygote resistant individuals were evaluated using the chi-squared test.

### Brush border membrane vesicle (BBMV) preparation

Individual (for linkage experiments) or pooled (for binding assays) guts dissected from fourth instar *S. frugiperda* larvae were used for preparation of BBMV by the differential centrifugation method^33^ as previously modified^34^. The final BBMV pellet was resuspended in ice-cold PBS solution (2 mM KCl,135 mM NaCl, 1.7 mM KH_2_PO_4_,10 mM Na_2_HPO_4_, pH 7.5) and total protein quantified using the Qubit fluorimeter using a protein assay kit (Invitrogen). The purity of the final BBMV preparations was determined using the enrichment in aminopeptidase-N (APN) and alkaline-phosphatase (ALP) activities compared to initial midgut homogenates as described elsewhere^35^. The APN and ALP activities were enriched 4–5 times in the final BBMV preparation compared to the initial midgut homogenates.

### Quantitative PCR

Relative quantification of *SfmALP2* transcript levels was performed as described previously^12^ using the Trizol reagent (Invitrogen) to extract total RNA from individually dissected midguts of 4th instar larvae. A cDNA template was then prepared from 2 µg of the total RNA with random hexamer primers using the High capacity cDNA reverse transcription kit (Invitrogen) according to the manufacturer’s instructions. Primers used for amplification of *SfmALP2* were (listed in 5′ to 3′ orientation) ALPFwd: GGCTTTCTGCCCAACTGT and ALPRev: TCTACGAGCCAATCAACG, while the ribosomal reference gene *SfL18* (GenBank AF395587.1) was amplified with primers SfL18Fwd: CGTATCAACCGACCTCCACT and SfL18Rev: AGGCACCTTGTAGAGCCTCA. Reactions were performed using 10-fold diluted cDNA from single midgut samples and using three technical replicates in an ABI 7900HT fast Real time PCR system (Applied Biosystems) on standard mode using the Quant6 studio flex manager software to collect the threshold cycle (Ct) values. The final reaction volume was 20 µl and included 200 ng of cDNA, 0.4 µM primers and 10 µl Power SYBR Green PCR Master Mix (Applied Biosystems). The cycling conditions included initial incubation of 55 °C for 2 minutes, and then denaturation at 95 °C for 10 minutes, followed by 40 cycles at 95 °C for 15 s, 55 °C for 30 s and 68 °C for 30 s, and a final step at 95 °C for 15 s. A single amplification peak was obtained for each reaction from the dissociation curves. Amplification efficiencies were greater than 90 percent for all the primer pairs used. Normalization of the relative transcript level was done using the *Sf L18* levels and calculating the relative *SfmALP2* transcript levels using the 2^−ΔΔCt^ method^36^. Relative expression was standardized considering the gene transcript levels detected for the Benzon strain as 1.

### Protein domain prediction

The ORF and amino acid sequence of the SfABCC2 protein were predicted using the ORF finder software (http://www.ncbi.nlm.nih.gov/gorf/gorf.html) and the online protein translation tool at the ExPASy Bioinformatics resource portal (http://web.expasy.org/translate/), respectively. The transmembrane topology was predicted using the Phobius web server^37^, and the ATP binding cassettes were predicted using a BLAST search of the Conserved Domains Database in NCBI (http://www.ncbi.nlm.nih.gov/cdd).

### Cry toxin binding assays

After thawing on ice, cell pellets were suspended in 300 µl of PBS pH 7.4 buffer and total protein quantified using the Qubit fluorometer (Invitrogen). All cell samples were diluted to the same concentration (4.97 mg/ml) with PBS pH 7.4 buffer. Binding experiments were performed as described elsewhere^12^ using 3.8 nM^125^I-Cry1Fa and 99.4 µg of Sf9 cell proteins in 100 µl final volume of PBS pH 7.4 plus 0.1% BSA. For competition assays, reactions included increasing concentrations of unlabeled Cry1Fa, Cry1Ab or Cry1Ca toxins. Two technical replicates were performed for each of the two biological replicates tested. The KELL software package (Biosoft, Cambridge, United Kingdom) was used to obtain representative binding affinity constant (*Kd*) and concentration of receptors (*Bmax*) values for toxins displacing Cry1Fa toxin binding to the cells.

### Cytotoxicity Assays

After 24 hours post-infection with recombinant virus, the infected cells were diluted 1:1 in fresh media containing Cry toxins at a 0.1 μg/ml final concentration. All treatments were performed in duplicate. The tubes were placed on a wheel and incubated for 24 hours at 27 °C. The following day, 30 μl aliquots of the treatments were diluted with an equal volume of 0.4% trypan blue dye and viability determined using a Countess automated cell counter (Invitrogen).

### Genotyping assays

The origin and number of the archived and recent field collected samples analyzed are shown in Table S1. Moths were captured using sex pheromone baited traps^38^ and collections extended over 7 days at sites near corn or cotton plantings to optimize trap capture efficiency of corn strain males. The collected specimens were identified as *S. frugiperda* by morphology then stored in a −20 °C freezer until required for analysis. Genomic DNA was isolated from individual carcasses of fifth instar larvae or adults using the Pure Link Genomic DNA mini kit (Invitrogen), following manufacturer’s protocols, and then quantified using a nanodrop spectrophotometer. Reactions for the Taqman® custom SNP Genotyping assay (Invitrogen) had a final volume of 10 µl in wells of a Micro Amp Fast optical 96 well reaction plate (Applied Biosystems) and followed manufacturer’s instructions. Reactions included 10–20 ng of genomic DNA as template, a VIC-labeled probe specific to the *SfABCC2mut* allele (5′ AAGCACATCGCCCACTT 3′), a FAM-labeled probe specific to the *SfABCC2* allele (5′ CCAAGCACATCCCACTT 3′), and the forward (5′ TGGAGGCCGAAGAGAGACA 3′) and reverse (5′AGGAGTTGACTGACTTCATGTACCT3′) primers. The plate was run in the Quant studio 6 Real Time PCR instrument (Applied Biosystems) using the following conditions: pre read stage at 60 °C for 30 seconds, hold stage at 95 °C for 10 minutes, PCR stage at 95 °C for 15 seconds and 60 °C for 1 minute for 40 cycles, post read stage at 60 °C for 30 seconds. The fluorescence in each well was measured in the post read stage of the PCR, and the software generated an allelic discrimination plot based on the post amplification intensity of the fluorescent probes.

Frequency of the resistant *SfABCC2mut* allele was determined using the Hardy-Weinberg equation under the assumption that selection was not present after removal of TC1507 from Puerto Rico in 2007^26^. We used the formula: F = (2 × ObsAa + Obsaa)/[2 × (ObsAA + ObsAa + Obsaa)]; where “F” is the frequency of the allele “a” (*SfABCC2mut*) and “Obs” the observed frequency of each of the three possible genotypes.

### Statistical analyses

Significant differences in *SfmALP2* transcript levels among individual larvae in linkage analyses were examined for means from three technical replicates for each sample using One Way ANOVA and post-hoc Holm-Sidak multiple comparison tests with *P* < 0.01 considering transcript levels detected for the 456 sample as reference. Similarly, significance of differences in Sf9 cell mortality in response to treatment with toxins or control buffer were tested considering values from two replicated assays using cells from two independent transfections (n = 2) using One Way ANOVA and post-hoc Holm-Sidak multiple comparison tests with *P* < 0.01.

### Data availability

The raw data used to prepare the figures and that support the findings of this study and the supplementary information are available from the corresponding author upon reasonable request.

## Electronic supplementary material

Supplementary information 1
