# Supplementary material for: Mechanism and DNA-based detection of field-evolved resistance to transgenic Bt corn in fall armyworm (Spodoptera frugiperda)
Source: Sci Rep. 2017 Sep 7;7:10877. doi: 10.1038/s41598-017-09866-y (PMC5589895; doi:10.1038/s41598-017-09866-y)
Supplement: Supplementary file 1 — Supplementary information 1 [file 41598_2017_9866_MOESM1_ESM.doc]

Supplementary Information

Mechanism and DNA-based detection of field-evolved resistance to transgenic Bt corn in fall armyworm (*Spodoptera frugiperda*)

Rahul Banerjee^1^, James Hasler^2^, Robert Meagher^3^, Rodney Nagoshi^3^, Lucas Hietala^4^, Fangneng Huang^5^, Kenneth Narva^2^, and Juan Luis Jurat-Fuentes^1,5*^

^1^Genome Science and Technology Program, University of Tennessee, Knoxville, TN 37996, USA

^2^Dow AgroSciences, Indianapolis, IN 46268, USA

^3^Behavior and Biocontrol Unit, USDA-ARS, Gainesville, FL 32608, USA

^4^Department of Entomology and Plant Pathology, University of Tennessee, Knoxville, TN 37996, USA

^5^Department of Entomology, Louisiana State University Agricultural Center, Baton Rouge, LA 70803, USA


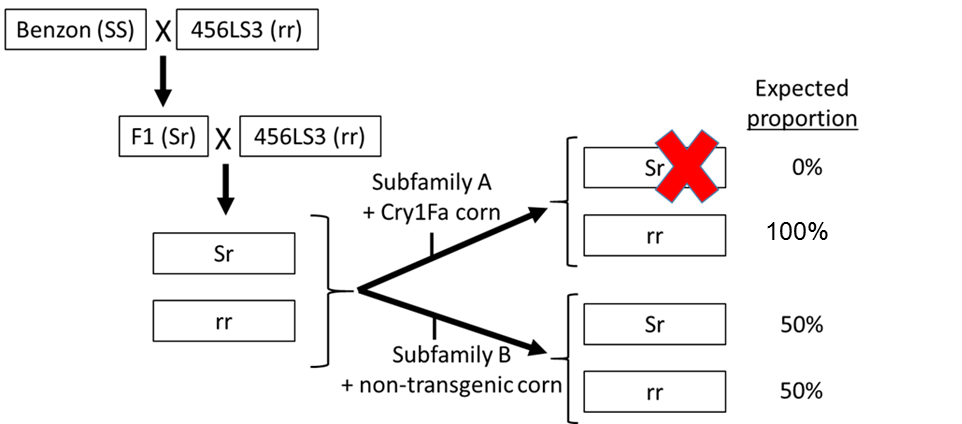


**Figure S1.- Diagram representing the backcrossing strategy followed to test for linkage between reduced SfmALP2 levels or the *SfABCC2mut* allele and resistance to transgenic corn event TC1507 producing the Cry1Fa protein**. Shown are the expected proportions of each genotype surviving the selection regime for each subfamily, assuming Mendelian inheritance of a single autosomal recessive resistance gene.

SfABCC2 ATGATGGACAAATCTAATAAAAATACCGCGGCAAATGGCAATGGTGGTCAACGTGCGGGC 60

SfABCC2mut ATGATGGACAAATCTAATAAAAATACCGCGTCAAATGG---------CACGGCTGTGGGC 51

****************************** ******* ** ****

SfABCC2 GAACCAAAGGAGAGAGTAAGAAAGAAACCAAACATATTGTCACGAATATTCGTTTGGTGG 120

SfABCC2mut GAACCAAAGGAGAGAGTAAGAAAGAAACCAAATATATTGTCACGTATATTCGTTTGGTGG 111

******************************** *********** ***************

SfABCC2 ATATTCCCTGTGCTCATCACTGGCAACAAGCGAGATGTTGAAGAAGACGATTTAATTGTT 180

SfABCC2mut ATATTCCCTGTGCTCATTACTGGCAACAAGCGTGATGTAGAAGAAGACGATTTAATTGTT 171

***************** ************** ***** *********************

SfABCC2 CCGAGTAAAAAATTCAATTCTGAAAGACAAGGAGAATATTTTGAAAGATATTGGTTCGAG 240

SfABCC2mut CCGAGTAAAAAATTCAATTCTGAAAGACAAGGAGAATATTTTGAAAGATATTGGTTCGAG 231

************************************************************

SfABCC2 GAGGTAGCAATTGCTGAGAGAGAAGACCGAGATCCTTCGTTATGGAAAGCGATGCGCCGC 300

SfABCC2mut GAGGTAGCAATTGCTGAGAGAGAAGACCGAGATCCTTCGTTATGGAAAGCGATGCGCCGC 291

************************************************************

SfABCC2 GCTTACTGGCTGCAATACATGCCGGGAGCTATCTTCGTGCTGCTCATTTCTGGATTAAGG 360

SfABCC2mut GCTTACTGGCTGCAATACATGCCGGGAGCTATCTTCGTGCTGCTCATTTCTGGATTAAGG 351

************************************************************

SfABCC2 ACAGCTCAGCCATTGCTGTTCTCTCAGCTCCTGTCCTACTGGTCGGTGGACAGCGAGATG 420

SfABCC2mut ACAGCTCAGCCATTGCTGTTCTCTCAGCTCCTGTCCTACTGGTCGGTGGACAGCGAGATG 411

************************************************************

SfABCC2 TCGCAGCAGGATGCTGGTCTCTATGCCCTGGCTATGTTGGGGATCAACTTCATAACCATG 480

SfABCC2mut TCGCAGCAGGATGCTGGTCTCTATGCCCTGGCTATGTTGGGCATCAACTTCATAACCATG 471

***************************************** ******************

SfABCC2 ATGTGCACACACCACAACAACTTGTTCGTCATGCGGTTCAGTATGAAAGTCAAGATCGCC 540

SfABCC2mut ATGTGCACACACCACAACAACTTGTTCGTCATGCGGTTCAGTATGAAAGTCAAGATCGCC 531

************************************************************

SfABCC2 GCCTCGTCACTCTTATTTAGAAAGTTGCTCCGCATGAGTCAAGTGTCAGTGGGCGACGTC 600

SfABCC2mut GCCTCGTCACTCTTATTTAGAAAGTTGCTCCGCATGAGTCAAGTGTCAGTGGGCGATGTC 591

******************************************************** ***

SfABCC2 GCCGGTGGAAAGTTAGTGAACTTGCTGTCCAACGATGTCGCGAGGTTTGACTATGCGTTC 660

SfABCC2mut GCTGGTGGAAAGTTAGTGAACTTGCTGTCTAACGATGTCGCAAGGTTTGACTATGCGTTC 651

** ************************** *********** ******************

SfABCC2 ATGTTCCTACACTACCTGTGGGTGGTGCCGCTGCAAGTCGGAGTCGTCCTGTACTTCGTG 720

SfABCC2mut ATGTTCCTACACTACCTGTGGGTGGTGCCGCTGCAAGTCGGAGTCGTCCTGTATTTCGTG 711

***************************************************** ******

SfABCC2 TACGATGCTGCTGGATGGGCGCCATATGTCGGTCTCTTTGGAGTCATCATTTTAATCATG 780

SfABCC2mut TACGATGCTGCTGGGTGGGCGCCATATGTCGGACTCTTTGGAGTGATTATATTAATCATG 771

************** ***************** *********** ** ** *********

SfABCC2 CCACTTCAAGCCGGTCTAACAAAACTCACGGGTGTTGTGAGGCGGATGACAGCTAAGAGG 840

SfABCC2mut CCACTTCAAGCTGGTCTAACTAAGCTCACGGGTGTTGTGAGACGGATGACAGCTAAGAGG 831

*********** ******** ** ***************** ******************

SfABCC2 ACTGACAAAAGAATTAAGCTTATGAGTGAAATTATTAACGGTATACAGGTCATCAAAATG 900

SfABCC2mut ACTGACAAAAGAATTAAGCTTATGAGTGAAATTATCAACGGTATACAGGTCATCAAAATG 891

*********************************** ************************

SfABCC2 TACGCTTGGGAGAAACCCTTCCAATTGGTGGTGAAAGCAGCTCGTGCGTATGAAATGAGT 960

SfABCC2mut TACGCTTGGGAGAAACCCTTCCAATTGGTGGTGAAGGCAGCTCGAGCGTATGAAATGAGT 951

*********************************** ******** ***************

SfABCC2 GCTCTTAGGAAATCTATCTTCATCAGGAGCATGTTCCTCGGGTTCATGTTGTTCACTGAG 1020

SfABCC2mut GCTCTTAGGAAATCTATCTTCATCAGGAGTATGTTCCTCGGGTTCATGTTGTTCACTGAG 1011

***************************** ******************************

SfABCC2 CGAAGTGTCATGTTTCTGACTGTGCTGACTCTAGCGCTGACTGGAAACATGATTAGCGCC 1080

SfABCC2mut CGAAGTGTTATGTTTCTGACTGTGCTGACTCTAGCGCTGACTGGAAACATGATTAGTGCC 1071

******** *********************************************** ***

SfABCC2 ACTTTGATTTATCCCATCCAACAATACTTCGGTATTATCACAATGAACGTTACCCTTATT 1140

SfABCC2mut ACTCTGATTTATCCCATCCAACAGTACTTCGGTATTATCACAATGAACGTTACCCTTATT 1131

*** ******************* ************************************

SfABCC2 TTACCGATGGCGTTTGCAAGTTTCTCTGAGATGTTGATATCCTTGGAACGTATTCAGGGA 1200

SfABCC2mut TTACCGATGGCGTTTGCGAGTTTCTCTGAGATGTTGATATCCTTGGAACGTATTCAGGGA 1191

***************** ******************************************

SfABCC2 TTCCTTCTTTTGGATGAGCGTTCAGACATTCAAATTACACCAAAAGTGGTGAATGGTGCT 1260

SfABCC2mut TTCCTTCTTTTGGACGAGCGTTCAGACATTCAAATTACACCAAAAGTGGTGAATGGTGCT 1251

************** *********************************************

SfABCC2 GGAAGCAAATTGTTCAACAACTCCAAGAAGGAGGGAGGCCTCGAGACTGGCATTGTGCTG 1320

SfABCC2mut GGAAGTAAACTGTTCAACAATTCCAAGAAGGAGGGAGGTCTCGAAACTGGCATTGTGCTG 1311

***** *** ********** ***************** ***** ***************

SfABCC2 CCAACAAAATACTCACCCACCGAAGCGAATATTGCCAGACCCATGCAGGATGAGCCTAAC 1380

SfABCC2mut CCAACAAAATACTCACCTACTGAAGCGAATATTGCCAGACCCATGCAGGATGAGCCTAAC 1371

***************** ** ***************************************

SfABCC2 ATGGCCGACTATCCTGTGCAACTTAACAAAGTTAATGCAACATGGGCGGACCTCAATGAT 1440

SfABCC2mut ATGGCCGACTATCCTGTGCAACTTAACAAAGTTAATGCAACATGGGCGGACCTCAATGAT 1431

************************************************************

SfABCC2 AACAAAGAGATGACACTCAAGAATATATCTTTACGTGTTCGCAAAAATAAACTATGCGCT 1500

SfABCC2mut AACAAAGAGATGACACTCAAGAACATATCTTTACGTGTTCGCAAAAATAAACTATGCGCT 1491

*********************** ************************************

SfABCC2 GTTATTGGACCTGTGGGATCAGGAAAGACCTCTCTTCTTCAGCTCCTTTTGAGAGAATTG 1560

SfABCC2mut GTTATTGGACCTGTGGGATCAGGAAAGACCTCTCTTCTTCAGCTCCTTTTGCGAGAGTTG 1551

*************************************************** **** ***

SfABCC2 CCTGTAACTAGTGGTAACCTCAGCATATCTGGTACCGTATCCTACGCTAGTCAGGAGCCG 1620

SfABCC2mut CCAGTGACTAGCGGCAATCTCAGCATATCTGGTACCGTATCCTACGCTAGTCAGGAGCCT 1611

** ** ***** ** ** *****************************************

SfABCC2 TGGCTGTTCCCTGCGACCGTGCGGGAGAACATTCTGTTTGGTTTGGAATACAATGTCGCC 1680

SfABCC2mut TGGCTGTTCCCTGCGACTGTGCGGGAGAACATTCTGTTTGGTTTGGAATATAATGTCGCC 1671

***************** ******************************** *********

SfABCC2 AAATATAAAGAGGTTTGTAAAGTCTGCTCATTACTGCCAGACTTTAAGCAGTTCCCGTAC 1740

SfABCC2mut AAATATAAAGAGGTTTGTAAAGTCTGCTCATTACTGCCAGACTTTAAGCAGTTCCCGTAC 1731

************************************************************

SfABCC2 GGTGACTTGTCTCTGGTTGGGGAGCGAGGTGTGTCACTGTCTGGTGGTCAGAGGGCCAGG 1800

SfABCC2mut GGTGACTTGTCTCTGGTTGGGGAGCGAGGTGTGTCACTGTCTGGTGGTCAGAGGGCCAGG 1791

************************************************************

SfABCC2 ATCAATTTGGCCAGAGCTGTCTACCGCGAGGCTGATATTTACTTGCTTGACGATCCTCTA 1860

SfABCC2mut ATCAATTTAGCCAGAGCTGTCTACCGCGAGGCTGATATTTACTTGCTTGACGATCCTCTA 1851

******** ***************************************************

SfABCC2 TCGGCTGTAGACGCAAACGTCGGCAGGCAACTATTCGACGGCTGCATCAAAGGATACCTC 1920

SfABCC2mut TCGGCTGTAGACGCAAACGTCGGCAGGCAACTATTCGACGGCTGCATCAAAGGATACCTC 1911

************************************************************

SfABCC2 AGTGGCAAGACATGCATCCTGGTCACCCACCAAATTCACTACCTTAAAGCTGCAGACTTT 1980

SfABCC2mut AGCGGCAAGACATGCATCCTGGTCACCCACCAAATTCACTACCTTAAAGCTGCAGACTTT 1971

** *********************************************************

SfABCC2 ATTGTAGTCCTAAACGAGGGTTCCGTCGAAAATATGGGCTCCTATGATGAACTAATGAAA 2040

SfABCC2mut ATTGTAGTCCTAAACGAGGGTTCCGTAGAAAATATGGGCTCGTATGATGAACTAATGAAA 2031

************************** ************** ******************

SfABCC2 ACTGGAACGGAATTCTCGATGCTGCTCTCTGACCAAGCTAGTGAAGGCTCTGACACTGAC 2100

SfABCC2mut ACTGGAACGGAATTCTCGATGCTGCTCTCTGACCAAGCTAGTGAAGGCTCTGACACTGAC 2091

************************************************************

SfABCC2 AAAAAAGAACGGCCAGCAATGATGCGGGGAATATCAAAGATGTCAGTCAAGAGTGACGAT 2160

SfABCC2mut AAAAAAGAACGGCCAGCAATGATGCGAGGAATATCAAAGATGTCAGTCAAGAGTGACGAT 2151

************************** *********************************

SfABCC2 GAGGAAGGTGAGGAGAAGGTTCAAGTATTGGAGGCCGAAGAGAGACAGTCGGGCAGCCTG 2220

SfABCC2mut GAGGAAGGTGAGGAGAAGGTTCAAGTATTGGAGGCCGAAGAGAGACAGTCGGGCAGTCTG 2211

******************************************************** ***

STOP

SfABCC2 AAGTGG--GATGTGCTTGGGAGGTACATGAAGTCAGTCAACTCCTGGTGCATGGTGGTAA 2278

SfABCC2mut AAGTGGGCGATGTGCTTGGGAGGTACATGAAGTCAGTCAACTCCTGGTGCATGGTGGTAA 2271

****** ****************************************************

SfABCC2 TGGCATTCCTCGTGTTGGTGATCACGCAGGGTGCTGCCACCACTACCGACTACTGGCTTA 2338

SfABCC2mut TGGCATTCCTCGTGTTGGTGATCACGCAGGGTGCTGCCACCACTACCGACTACTGGCTTA 2331

************************************************************

SfABCC2 GTTTCTGGACTAACCAGGTGGATGGATACATACAAACTTTACCCGAAGGAGAAAGTCCAA 2398

SfABCC2mut GTTTCTGGACTAACCAGGTGGATGGATACATACAAACTTTACCCGAAGGAGAAAGTCCAA 2391

************************************************************

SfABCC2 ATCCTGAGTTGAACACTCAAGTCGGTCTATTAACCACGGGACAGTACCTCATTGTCCACG 2458

SfABCC2mut ATCCTGAATTGAACACTCAAGTAGGTCTGAATTCCACGGGACAGTACCTCATCGTGCACG 2451

******* ************** ***** ******************* ** ****

SfABCC2 GCAGCGTAGTACTAGCCATTATAATATTGACGCAAGTCAGAATACTTTCCTTCGTAGTGA 2518

SfABCC2mut GCAGCGCAGTACTAGCCATTATAATATTGACGCAAGTCAGAATACTTTCCTTCGTAGTGA 2511

****** *****************************************************

SfABCC2 TGACTATGCGAGCTTCGGAAAATCTTCATAACACCATTTACGAGAAATTGATAGTGGCTG 2578

SfABCC2mut TGACTATGCGAGCTTCGGAAAATCTTCATAACACCATTTACGAGAAATTGATAGTGGCTG 2571

************************************************************

SfABCC2 TAATGAGATTTTTCGATACCAATCCATCGGGTCGTGTCTTGAACAGATTCTCAAAAGATA 2638

SfABCC2mut TAATGAGATTTTTCGATACCAATCCATCGGGTCGTGTATTGAACAGATTCTCAAAAGATA 2631

************************************* **********************

SfABCC2 TGGGTGCAATGGATGAGCTGTTACCGCGAAGCATGTTGGAAACGGTTCAGATGTACCTGT 2698

SfABCC2mut TGGGTGCAATGGATGAGCTGTTGCCGCGAAGCATGTTGGAGACTGTTCAGATGTACCTGT 2691

********************** ***************** ** ****************

SfABCC2 CTTTAGCCAGTGTCCTTGTGCTGAACGCCATAGCCTTACCGTGGACATTGATACCCACCA 2758

SfABCC2mut CTCTAGCCAGTGTCCTTGTGCTGAACGCCATAGCCTTACCTTGGACATTGATACCCACCA 2751

** ************************************* *******************

SfABCC2 CTGTGTTAATGTTTATATTTGTATTCCTATTGAAGTGGTACATTAATGCCGCTCAAGCTG 2818

SfABCC2mut CAGTGTTGATGTTTATATTTGTATTCCTATTGAAGTGGTACATTAACGCTGCTCAAGCTG 2811

* ***** ************************************** ** **********

SfABCC2 TGAAACGATTGGAAGGAACTACTAAGAGTCCTGTGTTTGGAATGATCAACTCTACTATCT 2878

SfABCC2mut TGAAACGATTGGAAGGAACTACAAAGAGTCCAGTGTTTGGAATGATCAACTCTACTATAT 2871

********************** ******** ************************** *

SfABCC2 CGGGACTCTCCACCATAAGAAGTTCCAATTCTCAGGACCGACTTCTAAACTCATTTGATG 2938

SfABCC2mut CTGGACTCTCCACCATAAGAAGCTCCAACTCTCAGGACCGACTTCTAAACTCATTTGATG 2931

* ******************** ***** *******************************

SfABCC2 ATGCACAGAATCTCCATACCAGCGCTTTCTACACATTTTTGGGTGGTTCAACCGCATTCG 2998

SfABCC2mut ATGCACAGAATCTCCATACTAGCGCTTTCTACACGTTTTTGGGTGGTTCAACCGCATTTG 2991

******************* ************** *********************** *

SfABCC2 GTCTGTACCTGGACACCCTTTGTTTAATCTACCTCGGAATTATCATGTCCATCTTCATTC 3058

SfABCC2mut GTCTGTACCTGGACACCCTCTGTTTAATCTACCTCGGAATTATCATGTCCATCTTCATTC 3051

******************* ****************************************

SfABCC2 TTGGTGATTTCGGTGAGCTGATACCAGTTGGTAGCGTCGGTCTGGCCGTCAGTCAGTCCA 3118

SfABCC2mut TTGGTGATTTCGGTGAGCTGATACCAGTGGGTAGCGTTGGTCTGGCCGTCAGCCAGTCCA 3111

**************************** ******** ************** *******

SfABCC2 TGGTGCTGACCATGATGTTGCAAATGGCCGCCAAGTTCACTGCCGACTTCCTGGGACAGA 3178

SfABCC2mut TGGTGCTCACCATGATGTTGCAGATGGCCGCCAAGTTCACTGCCGACTTCTTGGGACAGA 3171

******* ************** *************************** *********

SfABCC2 TGACAGCCGTCGAGAGGGTACTGGAATACACCAAGCTACCTACCGAAGAAAACATGGAGA 3238

SfABCC2mut TGACAGCCGTCGAGAGGGTCCTGGAGTACACTAAGCTACCTACCGAGGAAAACATGGAGA 3231

******************* ***** ***** ************** *************

SfABCC2 CTGGACCGACAACGCCACCAAAGGGATGGCCAAGTGCTGGAGAGGTGACGTTCTCAAACG 3298

SfABCC2mut CTGGACCGACAACGCCACCAAAGGACTGGCCAAGTGCTGGAGAGGTGACGTTCTCGAACG 3291

************************ ***************************** ****

SfABCC2 TGTACCTCAAGTATTCTCCTGACGACCCACCTGTACTGAAGGACTTGAACTTTGCAATCA 3358

SfABCC2mut TGTACCTCAAGTATTCTCCTGACGACCCACCAGTACTGAAGGACTTGAACTTTGCAATCA 3351

******************************* ****************************

SfABCC2 AGAGTGGCTGGAAGGTCGGAGTAGTTGGCAGAACTGGTGCTGGCAAGTCCTCGTTAATAT 3418

SfABCC2mut AGAGTGGATGGAAGGTCGGAGTAGTTGGTAGAACTGGCGCTGGCAAGTCCTCGTTGATAT 3411

******* ******************** ******** ***************** ****

SfABCC2 CAGCTCTGTTCCGGCTCAGCGACATTACAGGCAGCATCAAAATTGATGGCCTCGATACTC 3478

SfABCC2mut CGGCTCTGTTCCGGCTCAGCGACATTACAGGCAGCATCAAAATTGATGGCCTCGATACTC 3471

* **********************************************************

SfABCC2 AAGGAATTGCCAAGAAGCTTTTGAGATCAAAAATATCAATAATTCCGCAAGAGCCAGTGT 3538

SfABCC2mut AAGGAATTGCCAAGAAGCTTTTGAGATCAAAAATATCAATAATTCCGCAAGAGCCAGTGT 3531

************************************************************

SfABCC2 TATTCTCTGCCTCGCTGCGTTACAATCTGGACCCATTCGACAACTACAACGATGAAGATA 3598

SfABCC2mut TGTTCTCTGCCTCGCTGCGTTACAATCTGGACCCATTCGACAACTACAACGATGAAGATA 3591

* **********************************************************

SfABCC2 TTTGGAGAGCATTGGAACAGGTGGAACTAAAGGAGAGTATACCAGCCCTCGATTACAAAG 3658

SfABCC2mut TTTGGAGAGCATTGGAACAGGTGGAACTAAAGGAGAGTATACCAGCCCTCGATTACAAAG 3651

************************************************************

SfABCC2 TGTCGGAAGGCGGCACCAACTTCTCAATGGGACAACGTCAGCTGGTGTGCCTGGCGCGCG 3718

SfABCC2mut TGTCGGAAGGCGGCACCAACTTCTCAATGGGACAACGTCAGCTGGTGTGCCTAGCGCGCG 3711

**************************************************** *******

SfABCC2 CCATCCTCCGCTCAAATAAAATTCTCATCATGGACGAAGCTACCGCTAACGTCGATCCTC 3778

SfABCC2mut CCATCCTCCGCTCAAATAAAATTCTCATCATGGACGAAGCTACCGCTAACGTCGATCCTC 3771

************************************************************

SfABCC2 AGACGGATGCTTTGATCCAGAAAACAATCCGTAAACAATTTGCAACGTGCACCGTGCTCA 3838

SfABCC2mut AGACGGATGCTTTGATCCAGAAAACAATCCGTAAACAATTTGCAACGTGCACCGTGCTCA 3831

************************************************************

SfABCC2 CGATCGCGCATCGACTGAATACCATTATGGATTCAGATCGAGTACTAGTCATGGACCAGG 3898

SfABCC2mut CGATCGCGCATCGACTGAATACCATTATGGATTCAGATCGAGTACTAGTCATGGACCAGG 3891

************************************************************

SfABCC2 GAGTGGCCGCGGAGTTCGACCACCCCTACATCTTGCTATCTAACCCCAATAGCAAGTTCT 3958

SfABCC2mut GAGTGGCCGCTGAGTTCGACCACCCCTACATCTTGCTATCTAACCCCAATAGCAAGTTCT 3951

********** *************************************************

SfABCC2 CCTCAATGGTGAAGGAAACAGGCGACAACATGTCCCGGATCTTGTTCGAAGTAGCCAAAA 4018

SfABCC2mut CCTCAATGGTGAAGGAAACAGGCGACAACATGTCCCGGATCTTGTTCGAAGTAGCCAAAA 4011

************************************************************

SfABCC2 CAAAATATGAAAGTGATTCCAAAACCGCTTAA 4050

SfABCC2mut CAAAATATGAAAGTGATTCCAAAACCGCTTAG 4043

*******************************

**Figure S2.- Alignment of the open reading frame sequences of the full length cDNAs encoding SfABCC2 and SfABCC2mut proteins**. The “GC” insertion at the 2,218 base pair that results in a frameshift and premature stop codon is indicated by a red arrow in the alignment, and the stop codon (TGA) has also been marked red.


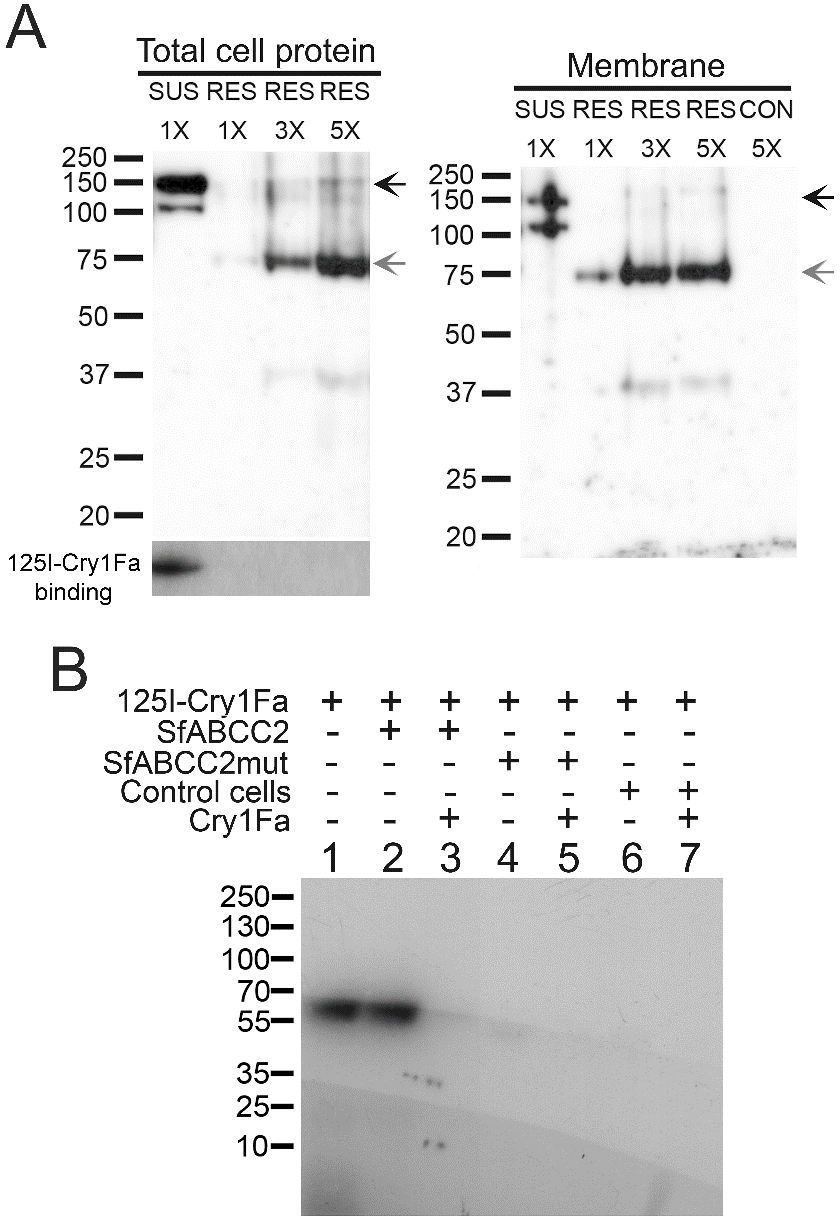


**Figure S3.-** **Expression of SfABCC2 and SfABCC2mut proteins in cultured Sf9 cells and Cry1Fa binding (A) and autoradiograph of ^125^I-Cry1Fa binding to these cells (B).** In A) production of recombinant SfABCC2 or SfABCC2mut proteins is detected by a His-tag present in the recombinant proteins. Shown is the detection of recombinant proteins in total cell protein extracts (Total cell protein) or purified membrane proteins (Membrane) samples from Sf9 cells producing SfABCC2 (SUS) or SfABCC2mut (RES) as well as control Sf9 cells (CON), as indicated. Relative protein loading levels for each lane (1X, 3X, or 5X) are indicated. Molecular weight markers are shown on the left for reference. Black arrow points to expected size of SfABCC2, while grey arrow indicates predicted molecular weight size for SfABCC2mut. Binding of ^125^I-Cry1Fa to each cell type is presented in the cropped bands below the “Total cell protein” blot. Components of binding reactions in B) are detailed above each lane: ^125^I-Cry1Fa toxin alone (lane 1), toxin bound to cells in the absence of unlabeled competitor (lanes 2, 4 and 6), and toxin bound in the presence of 500-fold unlabeled Cry1Fa toxin (lanes 3, 5 and 7). Cells in lanes 2 and 3 produced SfABCC2, cells in lanes 4 and 5 produced SfABCC2mut, and cells in lanes 6 and 7 were transformed with an empty vector (Control cells).


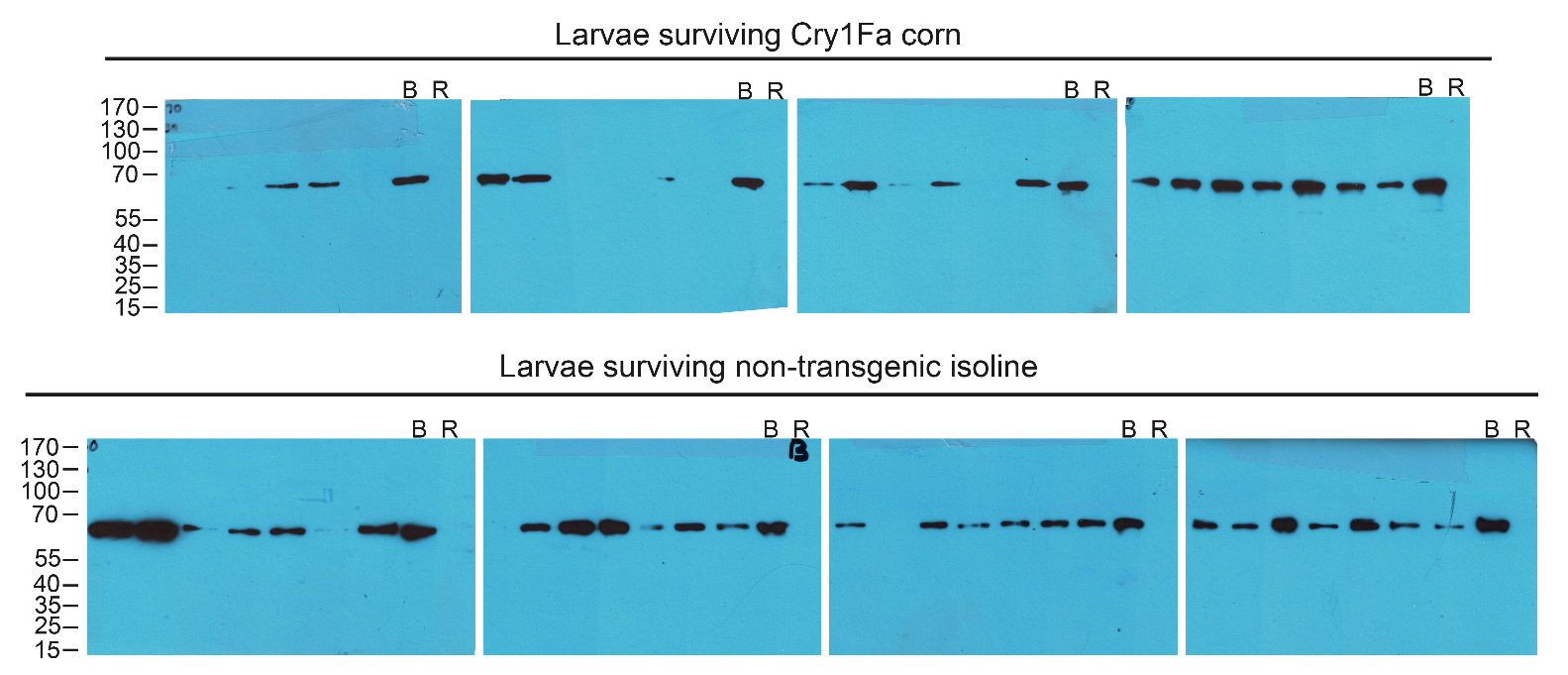


**Figure S4.- Detection of SfmALP2 in Western blots using specific antisera.** Shown are the full-length images from which the cropped areas shown in Figure 3B were obtained.


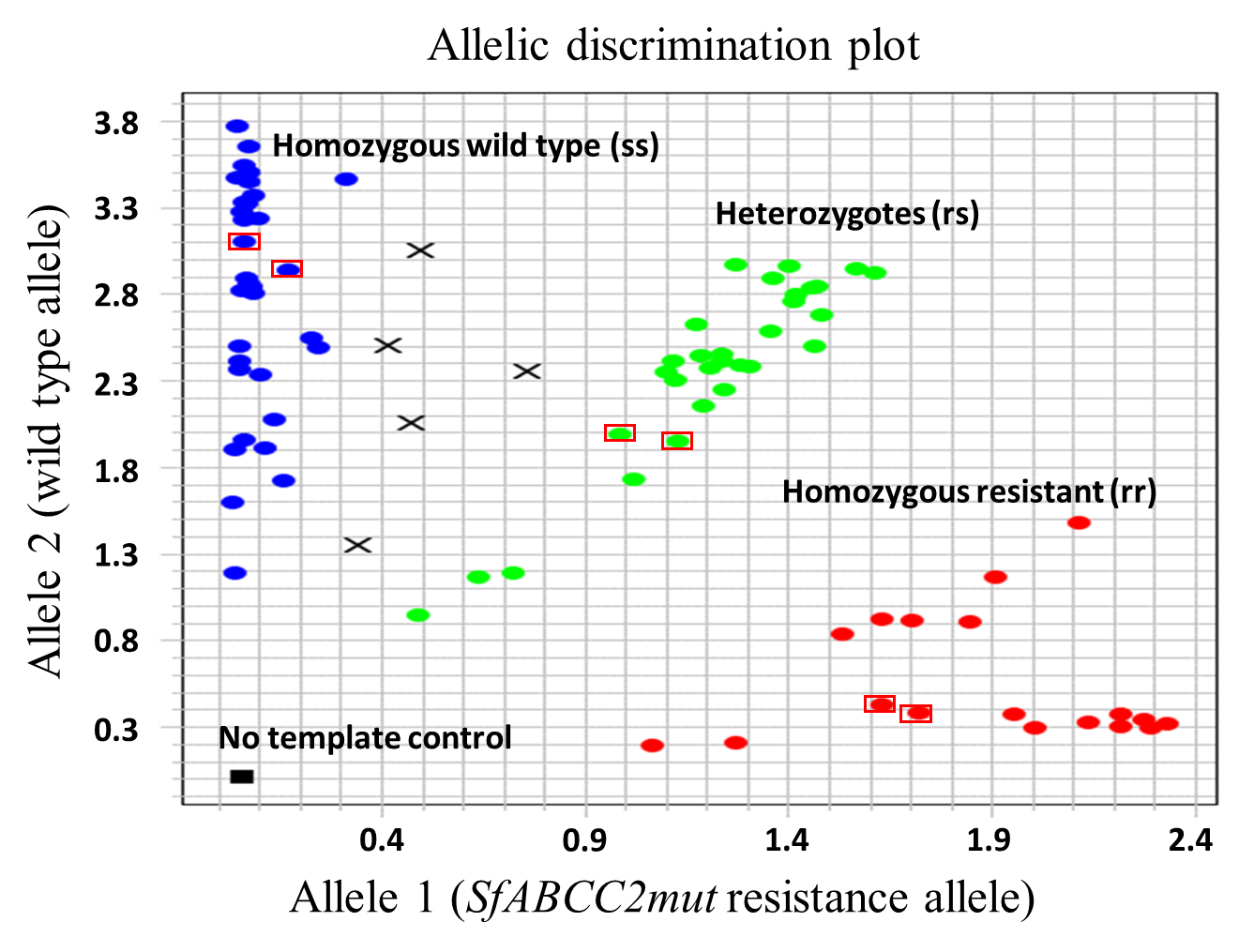


**Figure S5.- Allelic discrimination plot based on the post amplification intensity of the fluorescent probes in Taqman assays.** Each dot in the plot represents a single insect. Blue dots represent homozygous wild type, red dots are homozygous for the *SfABCC2mut* allele, and green dots represent wild type/*SfABCC2mut* heterozygotes. Internal controls for each genotype are indicated by the red rectangle. The no template control is given by the black box, samples with undetermined genotyped are denoted with an “X”.

**Table S1**.- Genotyping for the *SfABCC2mut* allele in archived and recently collected field samples. Shown are the location and date of collection, number of samples tested, and number of individuals with each genotype detected.

| **State/Country** | **County/Municipality** | **Date** | **Total** | **SS** | **Sr** | **rr** |
| --- | --- | --- | --- | --- | --- | --- |
| **Puerto Rico** | Isabela | 8/6/2007 | 24 | 24 | 0 | 0 |
|  |  | 8/20/2007 | 24 | 24 | 0 | 0 |
|  |  | 9/10/2007 | 24 | 24 | 0 | 0 |
|  |  | 9/17/2007 | 25 | 25 | 0 | 0 |
|  |  | 10/23/2007 | 24 | 22 | 0 | 2 |
|  |  | 10/30/2007 | 24 | 24 | 0 | 0 |
|  | Juana Diaz | 9/11/2007 | 24 | 24 | 0 | 0 |
|  |  | 9/18/2007 | 24 | 24 | 0 | 0 |
|  |  | 3/31/2009 | 116 | 67 | 40 | 9 |
|  | Salinas | 3/23/2017 | 76 | 34 | 26 | 16 |
| **Dominican Republic** | Santo Domingo | 6/30/2015 | 45 | 45 | 0 | 0 |
|  | La Vega | 8/8/2016 | 45 | 45 | 0 | 0 |
| **Florida** | Miami-Dade | 10/24/2012 | 27 | 27 | 0 | 0 |
|  |  | 6/20/2014 | 27 | 27 | 0 | 0 |
|  |  | 7/18/2014 | 27 | 27 | 0 | 0 |
|  |  | 8/19/2014 | 27 | 27 | 0 | 0 |
|  |  | 9/26/2014 | 27 | 27 | 0 | 0 |
|  |  | 11/7/2014 | 27 | 27 | 0 | 0 |
|  |  | 12/8/2014 | 27 | 27 | 0 | 0 |
|  |  | 12/19/2014 | 27 | 27 | 0 | 0 |
|  |  | 4/25/2016 | 13 | 13 | 0 | 0 |
|  | Palm Beach | 4/25/2016 | 13 | 13 | 0 | 0 |

**Materials and Methods for Supplementary Information**

Membrane protein purification

Aliquots (1.5 mg total protein) of control Sf9 cells transformed with an empty bacmid, and cells producing SfABCC2 or SfABCC2mut were used for purification of membrane proteins using the Mem-PER membrane protein extraction kit (Thermo Fisher), using manufacturer’s instructions. Final pellets containing membrane protein fractions were solubilized in 50 µl of electrophoresis sample buffer (50 mM TRIS, pH 6.8, 1.6% SDS, 7% glycerol, 4% β-mercaptoethanol, 0.016% bromophenol blue) and used for Western blotting without heat denaturing before electrophoresis to avoid formation of artefactual aggregates.

Western blotting

Proteins in BBMV (20 µg) for SfmALP2 detection were separated by SDS-10%PAGE electrophoresis, while proteins from Sf9 cell cultures (50 µg for 1X load) for detection of recombinant SfABCC2 were separated using precast Criterion AnyKd SDS-PAGE gels (BioRad). After electrophoresis proteins were electrotransferred at 4°C to a polyvinylidene difluoride (PVDF) filters in transfer buffer (25 mM Tris, 192 mM Glycine, 0.1% SDS and 20% methanol). The filters were blocked in blocking buffer (PBS pH 7.4 plus 0.1% Tween 20 and 3% BSA) for one hour at room temperature, and then were probed with rabbit antisera against SfmALP2 (1:5,000 dilution) overnight at 4°C or with anti-His antisera conjugated to horseradish peroxidase (1:10,000 dilution) for 1 h. at room temperature. The filters were then washed six times for 10 minutes each with washing buffer (PBS pH 7.4 plus 0.1% Tween-20 and 0.1% BSA). Filters for detection of recombinant protein production were then developed with enhanced chemiluminescence (Super Signal West Pico, Pierce) using a GE Imager 600 unit (GE LifeSciences). Filters for SfmALP2 detection were probed for 1 h at room temperature with goat anti-rabbit antisera conjugated to horseradish peroxidase (1:20,000 dilution). After washing, filters were developed using enhanced chemiluminescence and photographic film.

Cry toxin binding

1

For autoradiography of Cry1Fa binding, reactions included Sf9 cells (50 µg for 1X in Fig. S3A and 100 µg total protein for Fig. S3B) and 27.8 nM ^125^I-Cry1Fa in a final volume of 100 µl of binding buffer (PBS pH 7.5 plus 0.1% BSA). To determine non-specific binding, approximately a 200-fold excess of unlabeled Cry1Fa was included in the binding reactions. Reactions were allowed to progress for 1 hour at room temperature, and then stopped by centrifugation at 15,000 rpm for 10 min. at room temperature. Pellets were washed with 0.5 ml of binding buffer and the samples centrifuged as before. This washing process was repeated for a total of three times, and the final pellet was solubilized in 20 µl of electrophoresis sample buffer. Samples were then heat denatured for 10 min. at 95°C and loaded on a SDS-10%PAGE gel. After electrophoresis, gels were wrapped in Saran wrap and exposed to photographic film in a freezer.

**References cited**

1 Jurat-Fuentes, J. L., Gould, F. L. & Adang, M. J. Dual resistance t*o Bacillus thuringiens*is Cry1Ac and Cry2Aa toxins i*n Heliothis viresce*ns suggests multiple mechanisms of resistance*. Appl. Environ. Microbio*l**.** 69, 5898-5906 (2003).
